# S89. THE IMPACT OF COGNITIVE REMEDIATION ON COGNITIVE AND PSYCHOSOCIAL OUTCOMES IN SCHIZOPHRENIA AND THE ROLE OF INTRINSIC MOTIVATION

**DOI:** 10.1093/schbul/sby018.876

**Published:** 2018-04-01

**Authors:** Shayden Bryce, Jennie Ponsford, Stuart Lee, Eric Tan, Sean Carruthers, Richard Lawrence, Susan Rossell

**Affiliations:** 1 Monash University; 2 Monash-Epworth Rehabilitation Research Centre; 3 Monash Alfred Psychiatry Research Centre, The Alfred Hospital and Monash University, Central Clinical School; 4 Swinburne University, Monash Alfred Psychiatry Research Centre, The Alfred Hospital and Monash University; 5 Swinburne University and Monash Alfred Psychiatry Research Centre, The Alfred Hospital; 6 Swinburne University, Monash Alfred Psychiatry Research Centre, St. Vincents Mental Health

## Abstract

**Background:**

Cognitive remediation (CR) therapies are upheld as promising methods of reducing cognitive impairment in schizophrenia. However, controlled trials with blind assessors and active comparison conditions are lacking, along with evidence of generalization of CR to everyday function and self-efficacy. In addition, the role of patient-specific factors such as motivation in predicting adherence and training outcomes has not been investigated. This assessor-blinded, randomized controlled trial compared the impact of ‘drill-and-strategy’ CR with a computer game (CG) control delivered in a group-setting on cognitive function, independent living skills and self-efficacy, and examined the impact of intrinsic motivation on group attendance and treatment response.

**Methods:**

Fifty-six people with schizophrenia or schizoaffective disorder were randomized into CR or CG, and offered 20 one-hour sessions over 10 weeks. Measures of cognition (MATRICS consensus cognitive battery), psychopathology (Positive and Negative Syndrome Scale), self-efficacy (Revised Self Efficacy Scale) and independent living skills (Independent Living Skills Survey) were administered at baseline, end-group and three-months post-group. Intrinsic motivation (Intrinsic Motivation Inventory-Schizophrenia Research) was measured in-session at baseline and end-group.

**Results:**

Primary analysis was conducted for participants who completed end-therapy assessment (CR=22; Control=21). Linear mixed-effect analysis found a significant interaction effect for cognition (p=.028). Pairwise comparisons revealed that cognition was better at end-group and three-month follow-up than baseline for CR completers, with no differences between timepoints for controls. Three-quarters (77%) of CR completers showed a reliable improvement in at least one cognitive domain. A significant time effect was also evident for self-efficacy (p=.028), with the combined groups showing higher self-efficacy at end-group than baseline. No changes in independent living skills were observed. Early reports of program value predicted session attendance above baseline cognitive and clinical symptoms. Enhanced program interest and value over time increased the likelihood of reliable cognitive improvement.

**Discussion:**

Drill-and-strategy CR, delivered as a stand-alone treatment in a group setting, may improve cognition in schizophrenia when compared to active controls. Enhancing motivation may increase the likelihood of achieving meaningful cognitive improvements. This type of CR, however, may not translate to independent living domains, even if enhanced cognition and confidence in completing everyday behaviors is achieved. Independent living skills may need to be targeted directly to achieve meaningful changes in this domain.

